# Dental Times and Advertiser

**Published:** 1851-10

**Authors:** 


Dental Times and Advertiser.—This is the title of a new Quarterly Periodical, edited by Alfred A. Blandy, M. D., D. D. S., Baltimore, at the low price of $1 per annum. The number of the Times now before us presents a very neat appearance, containing some forty pages of reading matter, besides some eighteen or twenty pages of advertisements. It is to be issued intermediate with the Journal. We give it a hearty welcome, and will be glad to have it remind us every six weeks that the Journal, our old and established friend, is coming next.

